# Internal thoracic artery graft failure and recurrence of symptoms following single-vessel coronary artery bypass graft surgery

**DOI:** 10.1186/s13019-023-02384-1

**Published:** 2023-10-07

**Authors:** Mikael Janiec, Axel Dimberg, Rickard P. F. Lindblom

**Affiliations:** 1 Department of Cardiothoracic Surgery and Anesthesiology, Uppsala University Hospital, Uppsala, Sweden; 2 Department of Surgical Sciences, Uppsala University, Uppsala, Sweden; 3 Department of Cardiothoracic Surgery, Karolinska University Hospital, Stockholm, Sweden

**Keywords:** CABG, Coronary artery bypass grafting, ITA, Internal thoracic artery, Graft failure

## Abstract

**Objectives:**

Coronary events and disease recurrence following coronary artery bypass (CABG) surgery could derive from either failure in the internal thoracic artery (ITA) graft, failure in other conduits or progressive disease in the coronaries. We aim to estimate the contribution of ITA graft failure to the recurrence of symptoms after CABG surgery.

**Methods:**

Within the Swedish Web System for Enhancement and Development of Evidence-Based Care in Heart Disease Evaluated According to Recommended Therapies registry, we identified patients who had coronary artery bypass grafting from 1997 to 2020 with a single-vessel ITA graft bypass. Deaths, postoperative incidence of coronary angiography and the presence of a failed graft at the time of the angiography were recorded.

**Results:**

The study population consisted of 1939 patients with a mean follow-up time (SD) of 17.2 (5.6) years. The cumulative incidence (95% CI) at 20 years for a first clinically-driven postoperative angiography was 38.6% (36.2–41.1). A failed ITA graft was reported in 16.4% of the angiographies.

**Conclusions:**

A substantial part of recurrent symptoms of coronary artery disease do not seem to be related to ITA failure. Disease progression in the native coronary vessels may instead be the main driver of symptom recurrence.

## Introduction

The left internal thoracic artery (ITA) is regularly used for left anterior descending artery (LAD) grafting as it has demonstrated a better patency and confers a survival benefit compared to saphenous vein grafts (SVGs) [1]. Failure rates of ITA grafts has been reported to be 5—8% in early protocol angiography [2–4], and 15% at 10 years [2]. In clinically-driven catheterizations the rate seems to be as high as 27% [5], but the incidence of failing ITA grafts has been difficult to study and the prognostic factors and clinical implications at population level are not known. SVGs can develop symptomatic occlusions [6, 7], but most failures occur early after surgery without causing any symptoms and seem to have a limited influence on future cardiovascular events [8, 9]. Arterial graft failures may have a more negative impact than SVG failures on long-term survival [5, 10]. It is, however, often not be possible to establish in what way graft failure is related to symptoms leading to new coronary angiographies and interventions. The clinical importance of the failure of individual grafts in relation to progression of atherosclerotic disease in the coronary arteries is unknown and the over-all significance of graft failure could be overestimated. As arterial grafts have demonstrated to be associated with superior patency [2, 11], a considerable effort has been made to improve long-term graft patency results by using multiple arterial grafts [12–14]. A clinically significant improvement in outcome has, however, not been observed [15]. The contribution from the failure of the ITA-LAD graft to the recurrence of symptoms after CABG surgery is important to understand to what extent an improved patency of additional grafts can be expected to translate into superior clinical results.

We evaluate long-term outcomes in a large population of almost 2 000 patients operated with CABG with a single distal anastomosis using ITA as graft, in an observational study. By studying patients with a single coronary anastomosis, the relation between ITA graft failure and recurrence of angina symptoms can be more accurately assessed than in patients who received several grafts due to multiple-vessel disease. We aim to quantify the mortality, incidence of first angiography and evaluate the reported patency of the ITA graft in order to assess the significance of ITA graft failure for the long-term outcome.

## Patients and methods

### Data sources and study population

The study was approved by the Ethical Review Board of the Swedish Ethical Review Authority, Gothenburg, Sweden (Registration number 2020-06252). The Swedish personal identity number allows nationwide registries with the possibility of long-term follow-up. The Swedish Web System for Enhancement and Development of Evidence-Based Care in Heart Disease Evaluated According to Recommended Therapies (SWEDEHEART) registry [16] provides base-line data on all patients undergoing cardiac surgery in Sweden as well as records of all angiographies. Registered data includes the indication for the procedure as well as the finding of any failed grafts. Patients with a permanent residence in Sweden between 40 and 80 years of age with no congenital malformations or previous cardiac surgery, who underwent isolated CABG with a single distal anastomosis using the ITA as graft between 1997 and 2020, were identified within the SWEDEHEART registry. We obtained baseline characteristics at time of surgery and data on postoperative angiographies for included individuals. The government agency for official statistics (Statistics Sweden) provides aggregate mortality data for the entire population in Sweden, stratified for age and sex, for every year. By matching each individual in the study cohort to an estimated survival curve matched for sex, year of birth and year of surgery we calculate the estimated survival in a matched cohort in the general population.

### Outcomes

Date of death was obtained from the national population registry. The dates for CABG procedures and postoperative angiographies were available in SWEDEHEART. For each angiography the indication for the procedure and the presence of any significantly stenosed or occluded grafts is registered by the angiographer. The elapsed time from the operation to death and first angiography as well as the presence of a failed graft was used as endpoints. These outcomes represent robust, easily definable, clinically relevant events that are likely to have a high correlation with CAD symptoms and have been used previously to evaluate results after CABG [17].

### Statistical methods

Patient characteristics were described by using frequencies and percentages for categorical variables, and means and standard deviations for continuous variables. Outcome measures were evaluated in the population as the time from operation to death from any cause and time to first postoperative coronary angiography. Patients were followed from the date of surgery until the date of death from any cause or the end of follow-up (Sept 15, 2021).

The Kaplan–Meier method was used to illustrate cumulative survival and cumulative incidence of first postoperative angiography. Hazard functions were estimated for the risk of angiography and the risk of finding a failed ITA graft. Indications for the first angiography as well as the presence of failed grafts were described by using frequencies and percentages. Data management and statistical analyses were performed with the use of R version 3.1.3 (R Foundation for Statistical Computing, Vienna, Austria).

## Results

### Study population and baseline characteristics

In total, 1939 patients operated with CABG with a single distal anastomosis using the ITA as graft between 1997 and 2020 met all inclusion criteria. The mean follow-up time (SD) was 17.2 (5.6) years. Baseline characteristics for the study population are shown in Table 1.

**Table 1:** Baseline characteristics of included patients

Total study population	1939
Mean follow-up (years)	17.2 (5.6)
Mean (SD) age (years)	63.7 (9.5)
Female	585 (30.2%)
Mean (SD) body mass index (mg/kg2)	27.5 (8.2)
Body mass index not recorded	178 (9.2%)
Diabetes	346 (18.7%)
Presence of diabetes not recorded	85 (4.4%)
*Renal impairment*	
Normal, (CC > 85 ml/min)	814 (47.9%)
Moderately impaired (50–85 ml/min)	743 (43.8%)
Severely impaired (< 50 ml/min) off dialysis	132 (7.8%)
On dialysis	9 (0.5%)
Renal impairment not recorded	241 (12.4%)
COPD	73 (7.3%)
COPD not recorded	940 (48.5%)
Extracardiac arteriopathy	83 (8.3%)
Extracardiac arteriopathy not recorded	936 (48.3%)
Previous stroke	32 (3.7%)
Previous stroke not recorded	1077 (55.5%)
*Ejection fraction*	
Normal	204 (72.1%)
30–50%	71 (25.1%)
< 30%	8 (2.8%)
Ejection fraction not recorded	1656 (85.4%)
MI last 90 days	298 (29.7%)
Prior MI not recorded	934 (48.2%)
Previous PCI	384 (40.3%)
Previous PCI not recorded	985 (50.8%)
*Euroscore*	
0–2	671 (50.4%)
3–5	470 (35.3%)
> 5	191 (14.3%)
Euroscore not recorded	607 (31.3%)
30 day mortality	22 (1.1%)

*SD* standard deviation, *CC* creatinine clearence, *COPD* chronic obstructive pulmonary disease, *PCI* percutaneous coronary intervention, *MI* myocardial infarction

### Mortality

**Fig. 1:**
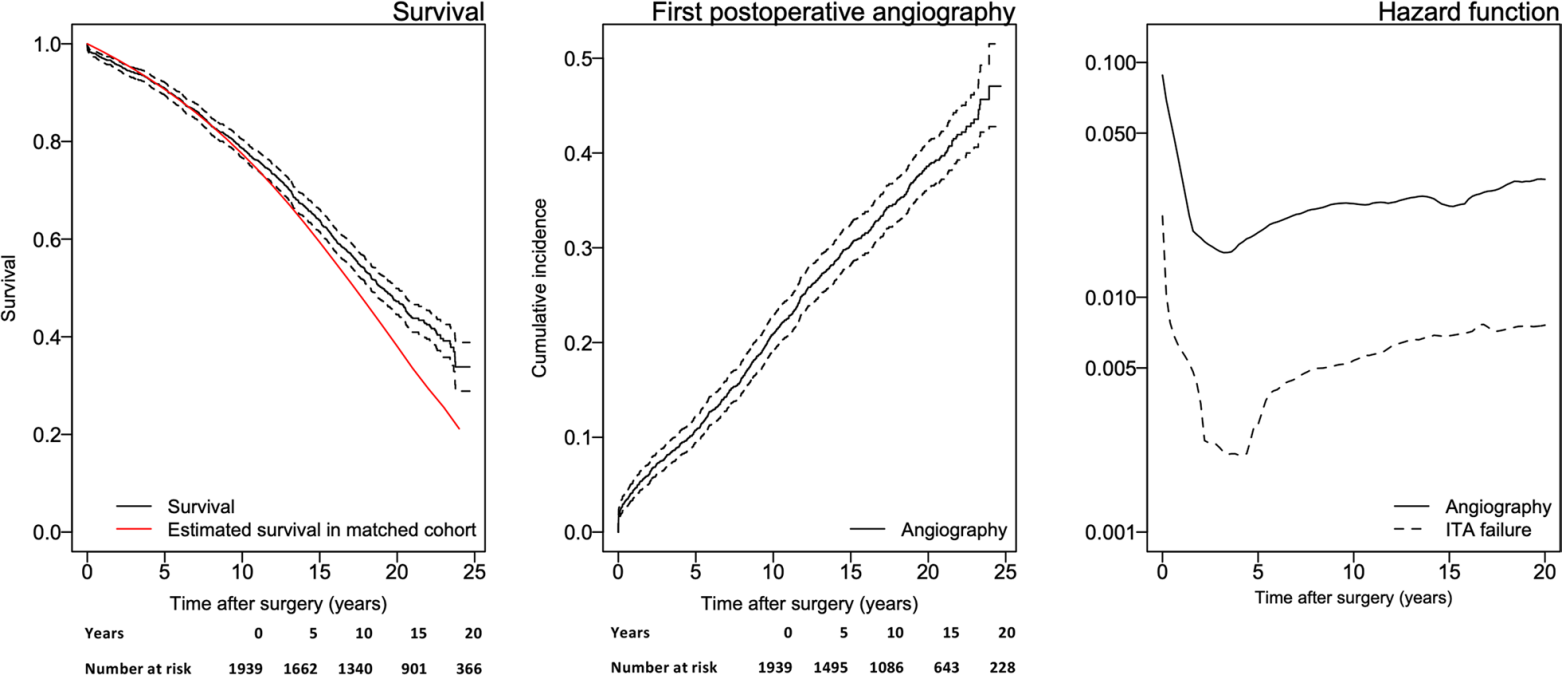
Kaplan–Meier estimates and 95% CIs of survival and cumulative incidence of first clinically-driven angiography for patients operated with CABG with a single distal anastomosis using the ITA as graft. Tables of number of patients at risk are also shown. Hazard rate for first angiography and for the risk of finding a failed ITA graft

Thirty-day mortality was 1.1% (22). Mortality occurred in 44.6% (865) of patients. Survival (95% CI) at 20 years was 47.2% (44.5–49.9) (Fig. 1). Expected survival for the same time period in an age and sex matched cohort from the general population was 38.1% (Fig. 1).

### Clinically-driven angiography

Postoperative clinically-driven angiography occurred in in 34.6% (671) of patients. The cumulative incidence (95% CI) at 20 years for the first clinically-driven postoperative angiography was 38.6% (36.2–41.1) (Fig. 1). The risk of angiography had a peak in the first months after surgery, was at its lowest about 3 years after surgery and later increased with time (Fig. 1). Angiographies were due to ST segment elevation myocardial infarction in 6.1% (41), unstable angina or non-ST segment elevation myocardial infarction in 38.2% (256), stable angina in 45.3% (304), and other (valvular disease, arrhythmia, other, unknown, research) in 10.4% (70) of patients (Table 2).

**Table 2:** Patients that underwent angiography during follow-up

*Indication for angiography*			
STEMI	41 (6.1%)		
Unstable angina/NSTEMI	256 (38.2%)		
Stable angina	304 (45.3%)		
Other	70 (10.4%)		
*Failed ITA graft*	Yes	No	Unknown
All	110 (16.4%)	488 (72.7%)	73 (10.9%)
< 6 months postoperatively	13 (20%)	31 (47.7%)	21 (32.3%)
> 6 months postoperatively	97 (16.0%)	457 (75.4%)	52 (8.5%)
STEMI	5 (12.2%)	27 (65.9%)	9 (15.7%)
Unstable angina/NSTEMI	44 (17.2%)	192 (75.0%	20 (7.8%)
Stable angina	50 (16.4%)	221 (72.7%)	33 (10.9%)
Other	11 (15.7%)	46 (65.7%)	11 (15.7%)

Indications for the first angiography and the number of patients where a failed graft was registered for all angiographies, for procedures within and later than 6 months after surgery, and for different indications

*STEMI ST* segment elevation myocardial infarction, *NSTEMI* non-ST segment elevation myocardial infarction, *ITA* internal thoracic artery

### Failed grafts

A failed ITA graft was reported in 16.4% (110) of the angiographies, and in 10.9% (73) of cases graft status was unknown. For procedures performed within six months of CABG surgery 20.0% (13) reported graft failure and in 32.3% (21) of patients graft status was unknown (Table 2). The fraction of angiography procedures where a failed graft was found was similar for the different indications for the procedure (Table 2) and seemed to be stable during the follow-up period (Fig. 1). For a subset of 392 angiographies the target vessel of the graft was registered. The target vessel was LAD in 91.8% (360), a diagonal branch in 2.8% (11), and other vessels in 5.4% (21) of patients.

## Discussion

We present a large cohort of individuals operated with single vessel coronary bypass using the ITA as graft and report the probability of finding a failed graft found during clinically-driven angiography. The over-all survival was similar to the expected mortality of a matched cohort from the general population. The cumulative incidence of post-operative angiography was approaching 39% 20 years after surgery. The ITA graft was classified as significantly stenosed or occluded in 18% of examined patients with available information regarding graft status. Consequently, about 7% of all operated patients experienced a post-operative angiography within 20 years after surgery, where a failed ITA graft is found. The number of symptomatic failures is, however, probably considerably lower, as asymptomatic ITA failures have been reported to range between 5 and 15% [2–4].

Predictors of ITA graft failure found during early protocol angiography have been identified as target vessel stenosis of less than 75%, additional bypass graft to diagonal branch, and not having diabetes mellitus [10]. Although a considerable number of patients with failed grafts found during the angiography may have had a previous asymptomatic failure unrelated to the current symptoms, it could be argued this number might be somewhat lower in a cohort of patients with one-vessel disease than after the much more common multi-vessel CABG. With only one stenosed coronary vessel it is highly probable that symptoms preceding the bypass surgery are related to this, presumable highly significant, stenosis and that these symptoms are likely to return if the bypass would fail. There are also no other bypass grafts to diagonal, marginal or posterior branches that could outcompete an ITA-LAD graft or mitigate the effects if it fails.

For the large majority of patients in this study, recurrent symptoms appear completely unrelated to graft failure, highlighting the significance of atherosclerotic progression in the coronary arteries and the importance of optimal secondary prevention. If similar results could be found also in the setting of multi-vessel CABG it might provide one possible explanation why it has been difficult to demonstrate an improvement in clinical outcome in patients operated with multiple arterial grafts [15].

For single vessel disease, CABG is rarely performed if PCI is an available option. Although the ITA graft probably remains the most reliable alternative, the study does not inform on the choice of treatment in general. The study is limited by the availability, quality and completeness of the registry data. The only patients that are believed to have been lost to follow-up are individuals undergoing coronary angiography outside of Sweden. It is not possible to identify if the right or left ITA was used. There was no formal definition of an occluded or significantly stenosed graft, and it was completely operator dependent. The degree of obstruction of the target vessels is also unknown. Some of the early postoperative angiographies may have been part of a planned hybrid procedure of multi-vessel disease or due to an inability to find or to graft a target vessel during surgery, explaining the high proportion of angiographies performed early after surgery where the ITA graft was not investigated. The majority of the patients in the study had their procedure in the first 5–10 years of the study period, when anti-platelet therapy was not as protocoled as it is now.

## Conclusions

The current study precisely demonstrates the performance of ITA-LAD in a real world setting and delineates the performance of the optimal CABG graft. The study removes the uncertainties normally associated with the interpretation of results of CABG outcomes due to the presence of multiple conduits. The results underline that symptomatic ITA failure is relatively uncommon on a population level and that disease progression in the native coronary vessels instead could be the dominating driver of symptom recurrence. Likely, more focus should be given to improved secondary prevention and better long-term medical treatment of CABG patients.

## Data Availability

The data underlying this article were provided by SWEDEHEART by permission. Data will be shared on request to the corresponding author with permission of SWEDEHEART. Code will be made available from the corresponding author upon reasonable request.
